# Design and evaluation of a recombinant multi-epitope antigen for serodiagnosis of *Toxoplasma gondii* infection in humans

**DOI:** 10.1186/s13071-015-0932-0

**Published:** 2015-06-11

**Authors:** Khalid Hajissa, Robaiza Zakaria, Rapeah Suppian, Zeehaida Mohamed

**Affiliations:** Department of Medical Microbiology & Parasitology, School of Medical Sciences, Universiti Sains Malaysia, 16150 Kubang Kerian, Kelantan Malaysia; Biomedicine Program, School of Health Sciences, Universiti Sains Malaysia, 16150 Kubang Kerian, Kelantan Malaysia

**Keywords:** Assembly PCR, Epitopes, Synthetic gene, *Toxoplasma gondii*, USM.TOXO1

## Abstract

**Background:**

Serological investigation remains the primary approach to achieve satisfactory results in *Toxoplasma gondii* identification. However, the accuracy of the native antigen used in the current diagnostic kits has proven to be insufficient as well as difficult to standardize, so significant efforts have been made to find alternative reagents as capture antigens. Consequently, multi-epitope peptides are promising diagnostic markers, with the potential for improving the accuracy of diagnostic kits. In this study, we described a simple, inexpensive and improved strategy to acquire such diagnostic markers. The study was aimed at producing novel synthetic protein consisting of multiple immunodominant epitopes of several *T. gondii* antigens.

**Findings:**

To accomplish our goals, a single synthetic gene of approximately 456 bp, which encodes potential epitopes of *T. gondii* antigens, was successfully constructed using gene assembly PCR. The constructed gene was cloned into a pET32a expression vector and transformed into BL21 *E. coli*. The entire protein was successfully expressed and purified. Subsequently, the preliminary diagnostic performance of expressed protein was evaluated by developing IgG enzyme-linked immunosorbent assay (ELISA) and Western blot analysis using human sera. The results showed 100 % sensitivity and specificity.

**Conclusion:**

A purified protein expressing multi-immunodominant epitopes of *T. gondii* was generated. Further studies are required to evaluate the immunogenicity in animal models and to verify the immuno-reactivity of USM.TOXO1 as a diagnostic antigen.

## Findings

### Background

Toxoplasmosis is a widespread parasitic disease caused by the intracellular protozoan parasite *Toxoplasma gondii* [1, 2]. The primary infection in healthy individuals is usually asymptomatic but severe clinical signs can be associated with the disease in immunocompromised patients [3–5]. Therefore, the development of simple, rapid, and sensitive diagnostic tests for *T. gondii* identification is crucial to reduce the risk of the disease in such patients [6].

The serological investigation of *T. gondii* remains the primary approach to achieve satisfactory results [7, 8]; however, producing reliable reagents and standard antigens remains difficult and expensive [6]. Currently, the use of crude native antigens in diagnostic methods has an important effect on the standardization of diagnostic tests as well as on the price of these kits.

In this sense, it is assumed that replacing this antigen in all current diagnostic kits with standard reagents will achieve a highly sensitive and specific diagnostic assay [9], and therefore significant efforts have been made to identify alternative capture antigens. As a result, a multi-epitope-based antigen approach using software-based prediction tools and molecular techniques may provide a novel and alternative means of acquiring less expensive and more accurate diagnostic kits [6, 10]. Furthermore, experimental evidence suggests that application of peptide-based antigen can meet the demand of serological test standardization and increase the sensitivity and specificity of these tests [9, 11, 12]. Consequently, assays based on such antigens are expected to be more sensitive and easier to standardize. Multi-epitope antigen as a potential capture antigen has been evaluated in several studies for different pathogens [9, 12–16], including *T. gondii* infection [6, 10, 11, 17, 18].

This study aimed to construct a synthetic gene that encodes multi-immuno-dominant epitopes of three *T. gondii* antigens by simple, inexpensive, and improved strategy for design and construction of a multi-epitope gene for acquirement of novel and promising diagnostic marker and vaccine candidate.

### Methods

**Fig. 1:**
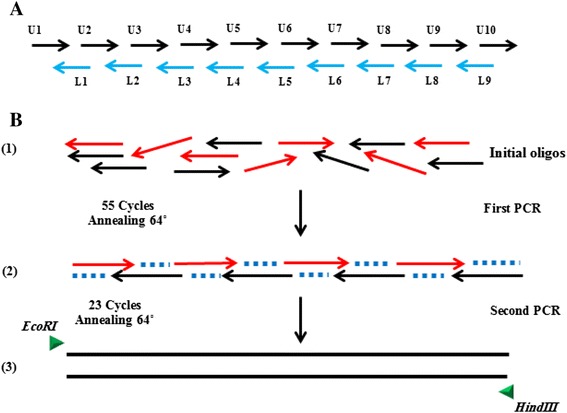
Schematic diagram of synthetic gene USM.TOXO1 construction; **a** Oligonucleotides design, U1–U10 is sense-strand primers. L1-L9 antisense-strand primers. **b** Assembly PCR steps *1*: Oligos mixture *2*: Gene assembly *3*: Gene amplification

Full amino acid sequences of SAG1, GRA2, and GRA7 were retrieved from the GenBank database. The immunodominant epitopes expressed within these antigens were identified by the ABCpred online prediction server [19]. Subsequently, three potential epitopes with high antigenicity and immunogenicity scores from each antigen were selected (Table 1). The epitopes were then combined in a manner that facilitated the design of the complementary oligonucleotides (Fig. 1). Finally, based on the DNA sequence of the predicted epitopes, a 456 bp synthetic gene (USM.TOXO1) was designed using VNTI computer program software (Life Technologies, USA).

**Table 1:** Amino acid sequence of 9 epitopes of three *T. gondii* antigen predicted by ABCpred

Gene	Predicted epitope	Sequence	Start position	Score
SAG1	SAG1_EP1	KLSAEGPTTMTLVCGK	202	0.91
	SAG1_EP2	AAVILTPTENHFTLKC	67	0.89
	SAG1_EP3	TEPPTLAYSPNRQICP	88	0.88
GRA2	GRA2_EP1	DERQQEPEEPVSQRAS	61	0.92
	GRA2_EP2	TQAPDSPNGLAETQAP	153	0.89
	GRA2_EP3	GVVNQGPVDVPFSGKP	28	0.87
GRA7	GRA7_EP1	AATASDDELMSRIRNS	26	0.93
	GRA7_EP2	MGLTRTYRHFSPRKNR	199	0.93
	GRA7_EP3	PELTEEQQRGDEPLTT	162	0.91

Subsequently, 19 overlapping, single-stranded DNA oligonucleotides (24–44 nt. in length) were designed and used to construct the USM.TOXO1 gene synthetically by assembly PCR, as described by Stemmer [20]. The synthetic gene was then cloned into a pET-32a expression vector (Novagen, USA). Consequently, the recombinant plasmid was transformed into *E. coli* BL21 (DE3) plysS competent cells (Novagen, USA). Following the confirmation of the sequences of inserts by DNA sequencing (IDT, Singapore), the protein expression was induced by isopropyl-D thiogalactopyranoside (IPTG) with a final concentration of 1 mM. Directly after purification of the synthetic protein by Ni-NTA column, SDS-PAGE and Western blot analysis were carried out to verify the expression of the candidate protein. In the western blot analysis, the purified protein was transferred to polyvinylidene fluoride (PVDF) membrane and then blocked with 3 % BSA in PBS for 1 h. The membrane was incubated with *T. gondii*-positive or negative human sera (1:100 dilution in blocking solution) or anti-histidine antibody (1:2500) for 1 h at room temperature. Bound antibodies were detected with HRP conjugated goat anti-human IgG and anti-mouse IgG (1:5000, Santa Cruz Biotechnology, Germany).

The preliminary integrity of the purified protein as a diagnostic marker was evaluated by immunoblotting and ELISA. For the ELISA analysis, diluted serum (1:100) from *T.gondii* sero-positive and sero-negative patients was used as the primary antibody. Briefly, 10 μg/ml of USM.TOXO1 synthetic protein was prepared in 100 μl of 0.05 M carbonate buffer (pH 9.6) and coated onto microtiter plates then incubated overnight at 4 °C. The plate was then washed (×3) with PBS-T for 5 min each time, the wells were blocked with PBS containing 3 % bovine serum albumin at 37 °C for 1 h, another three rounds of washes were carried out before the diluted human sera was added and incubated at 37 °C for l h. After three washings with PBS-T, a secondary anti-human IgG conjugated with horseradish peroxidase was added and incubated at 37 °C for 1 h. After final washing, TMB substrate was added and incubated for 15 min. The reaction was stopped by the addition of 2 M H_2_SO_4_. The optical density (OD) was then measured by using SpectraMax M Series Multi-Mode Microplate Readers (USA).

### Ethical approval

The study protocol was approved by Human Research Ethics Committee Universiti Sains Malaysia (HREC) (Approval number USM/JEPeM/15020034).

### Results

**Fig. 2:**
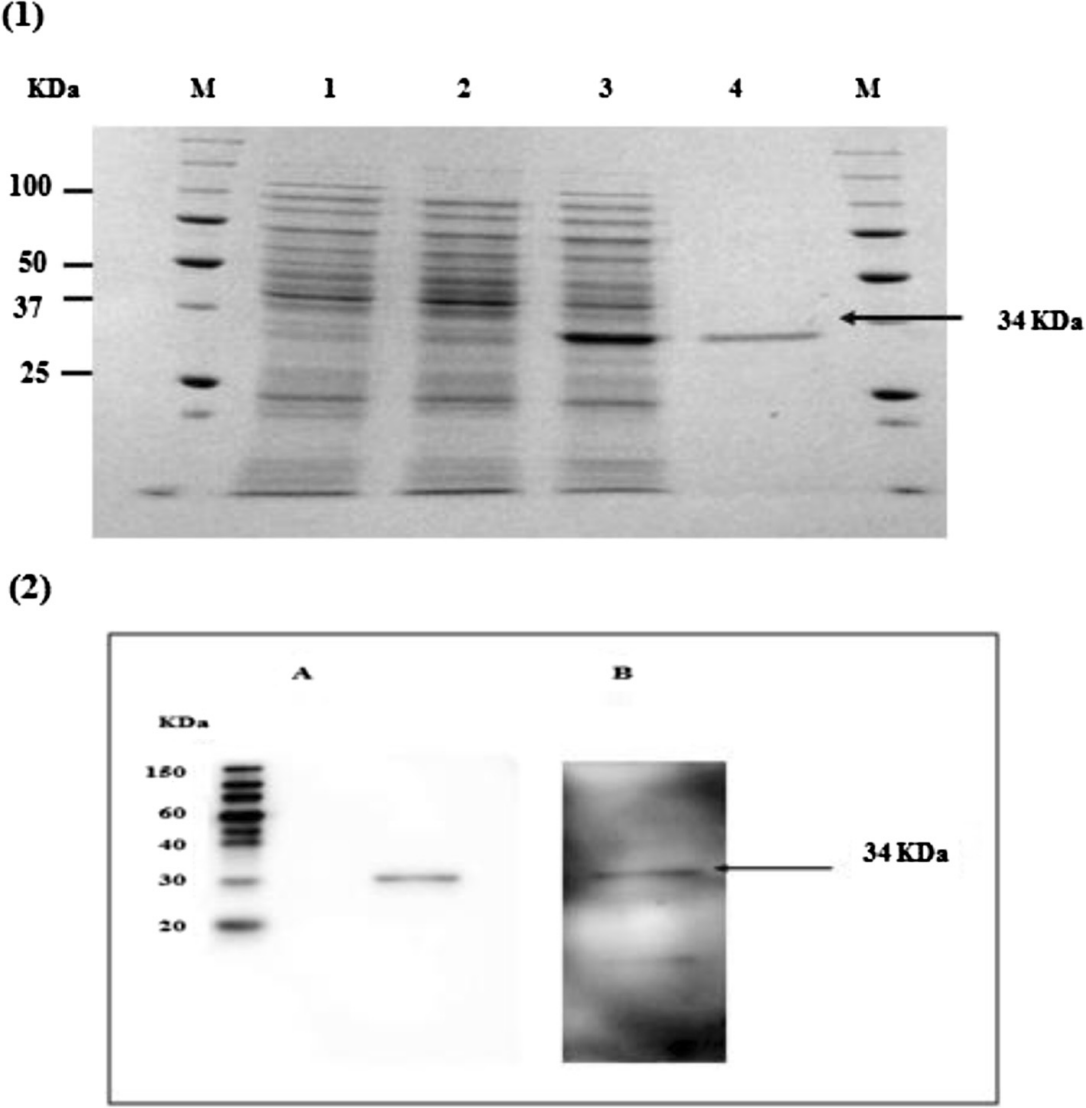
SDS-PAGE and WB Analysis of USMTOXO1 expressions in BL21 pLysS (DE3), **1** Coomassie blue stained; *Lane M*: molecular weight marker, *Lane 1*: lysate of IPTG-induced *E.coli* containing vector without insert, *Lane 2*: lysate of non-induced *E.coli* containing vector with insert, *Lane 3*: lysate of IPTG-induced *E.coli* containing vector with insert, *Lane 4*: purified USMTOXO1 synthetic protein **2** Western-blot analysis of purified USM.TOXO1; detected by (*A*) anti-His antibody (*B*) human sera

This study produced a single protein expressing nine immunodominant epitopes of *T. gondii* antigens. Briefly, USM.TOXO1 synthetic gene was designed using VNTI software based on the DNA sequence of the predicted epitopes. Subsequently, a mixture of 19 single-stranded DNA oligonucleotides was used in the assembly PCR reaction. A 456 bp synthetic gene was successfully amplified in the second PCR from the collection of DNA fragments generated by assembly PCR. Following the induction of the expression in BL21 *E. coli*, SDS-PAGE and Western blot analysis allowed successful identification of the purified synthetic protein (Fig. 2). The preliminary diagnostic performance of the developed ELISA, using 40 positive and 40 negative serum samples, showed highly immunoreactivity performance of these synthetic proteins.

### Discussion

Up to date, most of the current diagnostic kits use crude *Toxoplasma* antigens [6, 9, 21]. Unfortunately, application of tachyzoite crude antigen in diagnostic assays is not only associated with sensitivity and specificity problems but also with difficulties in standardization and cost effectiveness [9]. To overcome this limitation, epitopes can standardize diagnostic methods and increase the sensitivity and specificity of these tests [19, 22].

Bioinformatics tools are widely applied for epitope identification in protein analysis. Moreover, various epitope peptides are used to develop diagnostic antigen and epitope-based vaccines [6, 10, 14]. By using software-based prediction techniques, the epitopes of several *T. gondii* antigen were predicted and their antigenicity evaluated; for example, SAG1 [17] and GRA1 [10, 11, 18]. The newly synthesized immunodominant epitopes within *T. gondii* antigens SAG1, SAG2, SAG3, GRA5, GRA6, and P35 have the potential to be novel diagnostic antigens for the achievement of accurate diagnostic kits [6]. Moreover, peptide epitopes are effective in discriminating between recently acquired infection and past infection; thus, they can serve as effective tools for toxoplasmosis screening [6, 11].

High diagnostic performance can be achieved by using different epitopes as diagnostic markers. Alternatively, the use of a multi-epitope peptide that expresses a high density of conserved antigenic determinant can contribute to the achievement of a high degree of sensitivity and specificity [9].

Current attempts at producing epitope-based antigens are based on direct PCR amplification of single peptide for DNA template [9] chemical synthesis [10, 11, 14], splicing by overlap extension (SOEing) [16], or by synthesis of two complementary single-stranded DNA oligonucleotides based on the DNA sequences of the identified epitopes. The oligonucleotides will then anneal to generate double-stranded DNA that encodes for the predicted epitope. However, the use of this approach necessitates joining the individual peptide with a linker to construct multi-epitope peptides [6].

In general, the conventional production of multi-epitope peptides as mentioned above has been proven to be time-consuming, complex, and costly. In this study, assembly PCR was applied to generate a synthetic gene, a simple and cost effective method that relies on constructing a single synthetic gene encoding multi-immunodominant epitope peptides as a new strategy for developing effective and standard diagnostic markers [23]. Assembly PCR is a flexible technique that can be used for producing long DNA sequence from single-stranded oligos or a mixture of single and double-stranded DNA [20]. The approach is very interesting because overlapping regions can be joined without the need of DNA ligation. Interestingly, the most significant advantage of synthetic gene construction is that a DNA template is not required, which is particularly helpful if the organism of interest has no readily available sequence information.

To the best of our knowledge, the production of a single synthetic gene that encodes multi-epitopes of several *T. gondii* antigens was generated for the first time in this study. Thus, the USM.TOXO1 synthetic gene is the primary product of using assembly PCR to assemble different immunodominant epitopes from different antigens in a single protein. Therefore, assembly PCR appears to be very efficient in obtaining a synthetic protein that expresses a high density of conserved antigenic determinant that can contribute to achieving sensitive and specific serodiagnostic methods.

Given that the objective of producing multi-epitope antigens is to investigate their potential application in the detection of toxoplasmosis infection, the ability of USM.TOXO1 antigen to detect anti-*Toxoplasma* antibodies was tested by western blot and ELISA analysis. In addition, the results suggest that the immuno-reactivity of the synthetic protein as a capture antigen to specifically detect serum *T. gondii* antibodies is promising. The generated recombinant multi-epitope peptide is considered useful, and further investigation into the development of accurate diagnostic kits is recommended.

### Conclusion

In conclusion, the recombinant proteins containing different antigenic determinants of three *T. gondii* antigens were generated. Results suggested that the performance of the generated multi-immuno-dominant epitopes in serodiagnosis has great potential for the development of accurate diagnostic kits. The synthetic protein yielded better results; in that it promises the improvement of current diagnostic assay by applying such antigens. However, the limited serum samples used in this study are not sufficient to give a final assessment of diagnostic utility of this synthetic protein. Further investigation with large-scale sample sizes and IgM-positive serum is required.

## Consent

Written informed consent was obtained from the patient for the publication of this report and any accompanying images.
